# Unravelling the Adiponectin Hallmark and Exploring the Therapeutic Potential of Its Receptor Agonists in Cancer Metabolic Reprogramming

**DOI:** 10.3390/biom15060820

**Published:** 2025-06-05

**Authors:** Sanober Kafeel, Giuseppina Palmiero, Alessia Salzillo, Angela Ragone, Silvio Naviglio, Luigi Sapio

**Affiliations:** 1 Department of Precision Medicine, University of Campania “Luigi Vanvitelli”, 80138 Naples, Italy; sanober.kafeel@unicampania.it (S.K.); giuseppina.palmiero@unicampania.it (G.P.); alessia.salzillo@unicampania.it (A.S.); silvio.naviglio@unicampania.it (S.N.); luigi.sapio@unicampania.it (L.S.); 2 Department of Mechanistic Cell Biology, Max Plank Institute of Molecular Physiology, 44227 Dortmund, Germany

**Keywords:** adiponectin, metabolism, cancer, AdipoRon

## Abstract

As the most abundant fat-derived hormone, adiponectin plays an essential role in regulating energy homeostasis. Current evidence proposes the serum levels of adiponectin as a risk factor and a diagnostic/prognostic biomarker in cancer. Moreover, distinctive antineoplastic features have also been reported as a result of adiponectin supplementation in preclinical models. Mapping of the cancer-associated metabolic changes has elucidated a highly adaptable and interconnected system that allows malignant cells to sustain their growth and survival. Along with the pyruvate into acetyl-CoA conversion, downregulation of both lactate dehydrogenase and glycolysis-related genes depicts the main adiponectin-induced perturbations affecting glucose metabolism in cancer. Meanwhile, a multi-level approach involving lipid trafficking, catabolism, and de novo synthesis has been attributed to adiponectin in malignancies. The adiponectin receptor agonist AdipoRon has recently been recognized as a promising antineoplastic compound. Remarkably, AdipoRon-mediated changes in cancer metabolism occur together with its antiproliferative potential. This review aimed at recapitulating the modulatory effects of adiponectin, as well as those of its synthetic receptor agonists, in driving metabolic alterations in cancerous cells. A critical discussion is also conducted to deduce whether the adiponectin axis could serve as a putative target to address the metabolic reprogramming in cancer progression.

## 1. Introduction

The risk for comorbidities associated with overweight or obese conditions becomes dramatically escalated due to the establishment of various socio-economic factors that include a sedentary lifestyle, overeating, and poor dietary habits [1]. A growing number of studies attribute this risk to body fat accumulation, a manageable root cause for the onset of certain tumors [2]. Besides playing the conventional role of providing an additional reserve of energy, adipose tissue is a central endocrine organ, which secretes a wide variety of messengers collectively named adipokines [3]. Functional characterization of the adipose-derived mediators has highlighted prominent involvement in “metaflammation”, a type of low-grade chronic inflammation affecting both metabolic and immune responses [4]. However, the contribution from each adipokine to metaflammation is often diametrically opposite. Among the most abundant fat-derived cytokines, leptin endorses a pro-inflammatory state, while adiponectin usually displays anti-inflammatory features [5].

Cancer-related inflammation is currently recognized as a crucial environmental factor that fosters tumorigenesis and promotes the progression of malignant tumors [6,7]. At the same time, metabolic remodeling is constantly adopted by cancer cells to sustain unbridled growth and immune evasion [8,9]. Both inflammation and metabolic reprogramming are joined by an invisible thread; one feeds the other in a kind of vicious cycle [10]. Therefore, modulating adipokine signaling may reveal itself to be an attractive therapeutic target for constraining metaflammation in malignant disorders.

Several observational studies have shown that low serum levels of adiponectin are inversely associated with the risk of developing hormone-dependent tumors, as well as breast, cervical, endometrial, ovarian, and prostate cancers [11]. Current evidence also proposes the serum adiponectin as an independent diagnostic and prognostic biomarker of some tumors [12,13]. Additionally, adiponectin-mediated stimulation has been reported to counterbalance multiple oncogenic pathways, suppressing both cell growth and invasion [14,15]. AMP-activated protein kinase (AMPK) serves as the main intracellular target of adiponectin thanks to its ability to disrupt both metabolic checkpoints and cellular behavior [15]. Coincidentally, activators of AMPK like metformin and AICAR (5-aminoimidazole-4-carboxamide-1-β-d-ribofuranoside) have demonstrated an analogous outcome with respect to adiponectin [16,17]. There is no shortage of conflicting results concerning adiponectin-related antineoplastic features. Among them, angiogenesis remains one of the most controversial topics, since stimulatory and inhibitory effects have been ascribed to adiponectin in tumors [18,19,20].

Although these findings hint at the restoration of an adiponectin signature as a rational approach to reversing the malignant phenotype, different limitations have discouraged its translation in clinical practice [21]. Its heavy molecular mass and reduced half-life still remain an insurmountable challenge in administering this adipose-derived hormone therapeutically. The discovery of synthetic agonists has radically transformed the outlook in the last decade, reviving the possibility of targeting adiponectin signaling to treat cancer and other metabolic diseases [22,23]. AdipoRon is among the most widely studied adiponectin receptor agonists in oncology research. Mimicking the physiologic function of adiponectin, AdipoRon exhibited significant anticancer effects along with adaptive metabolic fluctuations in preclinical studies [24,25,26,27,28]. Several other synthetic agonists have been identified over the years, displaying beneficial effects in many chronic disorders via metabolite-sensing and signaling pathways [29,30].

This review aimed at recapitulating the modulatory effect of adiponectin in driving metabolic alterations in various cancer-stressed microenvironments. The implications of the newly emerged adiponectin receptor agonists in cancer metabolic reprogramming are also covered in this article. Lastly, targeting of adiponectin signaling is critically discussed as a complementary approach to rewiring metabolism in cancer cells.

## 2. Adiponectin

Adiponectin was first identified by Philipp Scherer as a 244-amino acid protein originating from the complement 1q family gene located on chromosome 3q27 [31]. The domains found in the genetic locus generate four different regions, as follows: (i) N-terminal signal peptide, (ii) variable domain (species-specific), (iii) collagen-like domain, and (iv) C-terminal globular domain, whose task is to recognize and bind membrane receptors [32]. Transcription and translation steps produce monomeric forms of adiponectin (full-length), which undergo post-transcriptional modifications that allow the formation of disulfide-linked oligomers composed of trimers (low molecular weight, LMW), hexamers (medium molecular weight, MMW), and multimers (high molecular weight, HMW) [33,34]. However, most of the circulating plasma adiponectin is constituted by medium- and high-molecular-weight forms [35]. LMW can also undergo proteolytic cleavage, resulting in a truncated form made up only by the C-terminal domain (globular) [36]. White adipose tissue is considered the predominant active endocrine site for adiponectin synthesis and secretion [32]. As the most abundant adipokine found in the bloodstream, adiponectin levels range between 5 and 50 μg/mL in healthy individuals, constituting almost 0.01% of total serum protein. Interestingly, serum levels of adiponectin were inversely correlated with body mass index (BMI) and insulin resistance [37].

Out of three adiponectin receptors identified so far, AdipoR1 (gene location chromosome 1q) and AdipoR2 (gene location chromosome 12p) belong to the seven-transmembrane domain receptor family [38]. AdipoR1 is predominantly expressed in several sites such as the skeletal muscle, heart, lungs, liver, kidney, and spleen. Contrastingly, AdipoR2 is found in higher aggregates in the liver and skeletal muscle [39]. The binding affinity for globular and full-length adiponectin differs between the two receptors. Typically, AdipoR1 shows high affinity for globular but very low affinity for full-length adiponectin, whereas AdipoR2 has an intermediate affinity for both globular and full-length forms [40]. The recent scientific literature confers an additional biological role to the AdipoRs, namely, that of evolutionarily conserved regulators of membrane homeostasis. Irrespective of the recognition of adiponectin, AdipoRs can promote the desaturation and incorporation of polyunsaturated fatty acids into the plasma membrane, thus regulating both composition and rigidity [41].

Adiponectin can also interact with T-cadherin, an atypical member of the cadherin superfamily that has lost transmembrane and cytosolic domains, preserving the glycosylphosphatidylinositol-anchored moiety [42]. Two different forms have been found in humans: a mature T-cadherin and a precursor harboring a pro-peptide within the five extracellular domains (ECs). EC1-EC2 are essential for the recognition of both MMW and HMW adiponectin, even though the presence of EC1-associated prodomain is preferred over the mature protein [43]. T-cadherin is highly expressed by endothelial and smooth muscle cells and, to a lesser extent, in the brain, lungs, and kidney. Although its intracellular signaling remains largely unknown, T-cadherin is featured by lateral movement within the plasma membrane that may affect the adiponectin interaction with AdipoR1/R2, thus modulating their cascade pathways [31]. Physiologically, T-cadherin is mainly involved in the adiponectin-mediated cardiovascular effects. In support of this claim, Denzel and co-authors reported how the heart recruitment of the circulating adiponectin is permitted by T-cadherin expression, which exerts protective effects in both cardiac hypertrophy and ischemia-reperfusion [44]. Concurrently, the genetic deficiency of T-cadherin increased the magnitude of damage after ischemia–reperfusion injury.

### 2.1. Physiological Role and Intracellular Signaling

Adiponectin is considered a well-known homeostatic regulator that encompasses a range of metabolic events affecting carbohydrates, lipids, and amino acids [36,45]. Variability in the expression of adiponectin receptors, as well as in their isoform affinity, ensures selective stimulation depending on the target tissue [46]. Besides affecting metabolic homeostasis, adiponectin is also involved in plenty of functions, which include inflammation, atherogenesis, and immune and neurological responses, among others [36].

AMPK and peroxisome proliferator-activated receptor alpha (PPAR-α) are widely recognized as the major intracellular mediators of adiponectin [14,47]. AdipoR1 principally acts via the activation of AMPK, while PPAR-α pathway predominantly becomes stimulated by the consequence of adiponectin binding with AdipoR2 [48]. As upstream activators of AMPK and PPAR-α, there is the adaptor protein phosphotyrosine interacting with PH domain and leucine zipper 1 and 2 (APPL1 and APPL2), which positively and negatively mediate adiponectin signaling, correspondingly [49]. APPLs also play a crucial role in enhancing insulin sensitivity by facilitating the binding of insulin receptor substrate proteins 1 and 2 (IRS 1/2) to the insulin receptor after either insulin or adiponectin stimulation [50].

The molecular mechanisms underlying the APPL1-mediated AMPK activation were firstly described in C2C12 muscle cells [51]. Deepa and coworkers demonstrated that adiponectin causes APPL1-induced protein phosphatase 2A (PP2A) stimulation, thereby preventing liver kinase B1 (LKB1) phosphorylation via the protein kinase Cζ (PKCζ). Loss of the phosphoryl group from serine 307 leads to LKB1 translocation into the cytosol, where it interacts with APPL1 by enabling AMPK. Besides APPL1, adiponectin can also modulate AMPK by acting on calmodulin-dependent protein kinase kinase (CaMKK-β) [52]. Both phospholipase C (PLC)-dependent and -independent calcium (Ca^2+^) release has been observed in the cytoplasmic compartment after adiponectin binding with AdipoR1. In myoblasts, for instance, adiponectin drives PLC to produce inositol triphosphate (IP3), which in turn stimulates IP3 receptors on the endoplasmic reticulum (ER), causing the cytosol accumulation of Ca^2+^ [52]. However, adiponectin can also promote Ca^2+^ mobilization from the extracellular pool by controlling entry cell capacity, as demonstrated in porcine aortic endothelial cells [53]. Irrespective of the source, the intracellular increase in this second messenger facilitates CaMKK-β activation, which contributes to sirtuin 1 (SIRT1) deacetylation and to AMPK phosphorylation. Among other residues, threonine 172 is critical to switching on the catalytic subunit of AMPK (AMPKα) and thus triggering the related signaling cascade [54]. However, as occurred for other kinases, two complementary effects contribute to keeping AMPK active along with threonine 172 phosphorylation: dephosphorylation by phosphatases and allosteric activation by AMP [55]. Coincidentally, rises in both AMP:ATP and ADP:ATP ratios equip AMPK to meet cellular demands in stress conditions.

**Figure 1:**
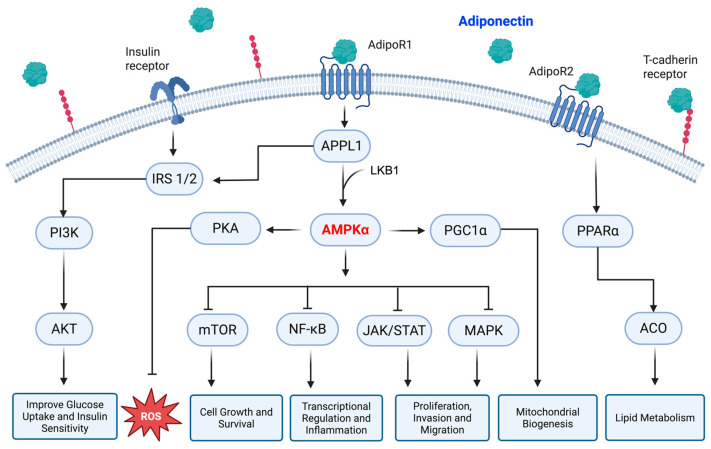
Schematic representation of the adiponectin-mediated intracellular pathways. Adiponectin mainly acts via AdipoR1 and AdipoR2 receptors, which in turn stimulate both AMPK and PPARα, respectively. AMPK further modulates several downstream signaling cascades, thus defining the cell’s fate. The APPL1-IRS 1/2 axis is responsible for adiponectin-induced insulin sensitivity instead. APPL1: PH domain and leucine zipper 1, IRS 1/2: insulin receptor substrate proteins 1 and 2, PI3K: phosphatidylinositol 3-kinase, AKT: protein kinase B, LKB1: liver kinase B1, AMPKα: AMP-activated protein kinase alpha, PKA: protein kinase A, ROS: reactive oxygen species, PGC1α: peroxisome proliferator-activated receptor gamma coactivator 1 alpha, mTOR: mammalian target of rapamycin, NF-kB: nuclear factor kappa B, JAK: Janus kinase, STAT: signal transducer and activator of transcription, MAPK: mitogen-activated protein kinase, PPARα: peroxisome proliferator-activated receptor alpha, ACO: acyl-CoA oxidase. Created in BioRender (https://BioRender.com, accessed on 29 May 2025).

Moving to the downstream effectors, adiponectin manipulates several signaling cascades that convey the information from the surface to the cell nucleus. A special mention should be made for the mammalian target of rapamycin (mTOR), p38 mitogen-activated protein kinases (MAPKs), phosphatidylinositol 3-kinase/protein kinase B (PI3K/AKT), nuclear factor kappa B (NF-κB), and Janus kinase/signal transducer and activator of transcription (JAK/STAT) as the main molecular interactors involved in adiponectin-mediated signaling transduction [56]. Principally, PI3K/AKT is involved in adiponectin-mediated insulin sensitization, since its recruitment by IRS 1/2 causes the synthesis of glycogen and glucose uptake along with the inhibition of lipolysis [47]. On the other hand, AMPK leads to IκBα dephosphorylation, thus preventing its degradation and the nuclear translocation of NF-κB [35]. Associated with the AMPK stimulation, adiponectin mediates the activation of protein kinase A (PKA), which enhances the formation of nitric oxide (NO) and negates the generation of reactive oxygen species (ROS) [46]. The higher ceramidase activity of adiponectin receptors also promotes the formation of sphingosine and free fatty acid [57]. The sphingosine obtained from this reaction can be further phosphorylated to produce sphingosine 1-phosphate (S1P), an essential signaling molecule in different cellular processes [58]. A schematic representation of the main intracellular signaling modulated by adiponectin is displayed in Figure 1 for easy reference.

### 2.2. Involvement in Malignant Diseases

Adiponectin has been recognized as an antineoplastic molecule in different malignant disorders, such as breast, colon, lung, thyroid, and endometrial cancers [59]. However, the current literature also contains many discrepancies about its malignant engagement (Table 1). The same adiponectin-mediated anticancer features have been ascribed to different mechanisms (Table 2), and some of them are discussed below.

Whilst lower serum levels of adiponectin correlate with a higher risk of breast cancer [60], treating breast cancer cells with the same adipose-derived adipokine provides conflicting results depending on estrogen receptor alpha (ERα) status [75,76]. Specifically, adiponectin has been proposed to stimulate tumor growth in ERα-positive MCF7 cells and simultaneously induce antiproliferative effects in ERα-negative MDA-MB-231 cells [77]. Regulation in cell cycle genes, as well as in PI3K/AKT and AMPK signaling, was used as supporting evidence for the adiponectin outcome in MDA-MB-231 cells [76]. The lack of susceptibility in ERα-positive cells was further corroborated by the use of second-generation peptides modulating adiponectin receptor responses [77]. Otvos and colleagues identified the autocrine production of adiponectin as the cause of cell growth induction in MCF7 cells treated with the adiponectin agonist peptide ADP400. Emerging data also proved that adiponectin behaves as a growth factor in ERα-positive obese breast cancer patients [78]. Although the exact explanation remains largely unknown, these findings emphasize how ERα signaling may enhance the potential inhibitory threshold of adiponectin. More generally, the interplay between adiponectin and growth factors continues to be uncertain in breast cancer and warrants further investigation [79].

The diagnostic utility of measuring adiponectin serum levels has revealed controversial results in lung cancer. Petridou and coworkers reported no changes in blood concentrations among healthy individuals in contrast to lung cancer patients, but significantly lower levels were observed in patients progressing towards advanced stages [62]. This association was denied by Karapanagiotou, who emphasized the lack of significant differences even in advanced non-small-cell lung cancer (NSCLC) patients [64]. More recently, Nigro and collaborators have displayed a marked reduction in adiponectin levels surrounding adenocarcinoma subtype and HMW oligomers [63]. Certainly, the histopathologic features of the enrolled population might play a critical role in explaining this different outcome. Taking into the account factors like age, sex, BMI, and tumor classification may clarify the diagnostic relevance of adiponectin in lung cancer. The proposed therapeutic role of adiponectin appears very promising for neoplasms affecting pulmonary parenchyma. One of the few conflicting reports dates back to 2010, when Denzel et al. demonstrated that the deficiency of adiponectin dysregulates vascularization and promotes pulmonary metastasis in transgenic mice [80]. Otherwise, the repressive effects of adiponectin on lung cancer have mainly been linked to the induction of ERK1/2 and AKT pathways [81]. The downregulation of cyclic AMP-responsive element-binding protein (CREB) was also identified to be involved in the antiproliferative effects of adiponectin in this kind of tumor [82]. More recently, the supplementation of adiponectin to NSCLC cells has been reported to alter the mesenchymal-to-epithelial transition, inhibiting both migration and invasion abilities [83]. We also demonstrated that the adiponectin receptor agonist AdipoRon impairs proliferation, viability, and stemness in NSCLC cells, slowing down cell cycle progression and triggering AMPK activation [28].

An increased risk of developing colorectal cancer because of the attenuated adiponectin levels has been supported by several studies [65,66,67]. Adiponectin has also proven to reduce cell proliferation, adhesion, and invasion in colorectal carcinoma effectively. AMPK activation has been established as essential for inducing the inhibitory effects of adiponectin in this intestinal tumor [84,85]. Correspondingly, apoptosis induction via extracellular signal-regulated kinase 1/2 (ERK1/2) and the cleavage of caspase 3 was the main adiponectin-induced mechanism suggested by Nigro and colleagues for restraining CaCo-2 and HCT116 cell growth [86]. An especially critical view of adiponectin has been disclosed by Ogunwobi, who proposed an active role of this adipose-derived cytokine in the regulation of gastrointestinal mucosal function, inflammation, and colon carcinogenesis [87]. This conflicting discrepancy might be connected to the availability of nutrients in tumor microenvironments, as adiponectin was found to promote colorectal cancer survival in glucose deprivation but produce inhibitory action in the presence of high glucose levels [88].

The underlying mechanisms by which circulating adiponectin affects cancer progression are poorly understood in thyroid cancer. However, individuals having a higher BMI and lower adiponectin index showed a positive correlation with the risk of developing this endocrine cancer [68]. On the contrary, a lack of association between serum adiponectin levels and medullary thyroid cancer was observed in a case–control study by Abooshahab and coworkers [69]. Two molecular mechanisms have emerged in the relationship between thyroid cancer and adiposity so far, namely, the phosphorylation of retinoblastoma protein (RB) and JAK2/STAT3 signaling expression [89,90]. On a cellular level, adiponectin binding to its receptors leads to AMPK phosphorylation, which results in both activation and/or inhibition of a whole signaling series. The most active anticancer pathways in adiponectin-mediated AMPK action include PI3K/AKT, ERK1/2, STAT3, β-catenin, ubiquitin carboxyl-terminal hydrolase 2 (USP2), and B-cell leukemia/lymphoma 2 (BCL-2) in this type of cancer [90]. A recent study also noted the impact of recombinant adiponectin in inhibiting cell growth and the metastatic potential of papillary thyroid cancer via the autophagy regulatory pathway [91].

Although there are some inconsistencies about the risk [72], lower adiponectin levels have been associated with more aggressive features in patients with endometrial cancer [70,71]. LKB1 is highlighted to be essential for adiponectin responses in endometrial cancer. As a substantial population of endometrial neoplasms harbors phosphatase and tensin homolog (PTEN) mutations, adiponectin might interact with PTEN through LKB1, thus impairing cell cycle regulation [71]. Remarkably, the knockdown of LKB1 eradicated adiponectin outcomes, restoring cell proliferation, clonogenic potential, and invasion in endometrial cancer. Adiponectin also impaired endometrial cancer cell growth by inducing the activation of AKT and diminishing cyclin D1 expression [92]. Interestingly, the activation of ERK1/2 signaling raised the incidence of endometrial lesions to at least two-thirds in adiponectin-null mice [93].

Among other cancers, there is weak evidence of an inverse association of adiponectin with the overall incidence of prostate cancer [74]. Nevertheless, the lower expression of adiponectin is more prominent in aggressive than the benign form of prostate cancer [73]. A study tested the efficacy of adiponectin through the use of the adiponectin receptor agonist ADP355, demonstrating inhibitory effects in a mouse model of prostate cancer-bearing subcutaneous LNCaP xenografts [94]. In vitro analyses demonstrated that the inhibitory response of adiponectin is facilitated by the blockage of both mTOR and vascular endothelial growth factor (VEGF) expression [95].

**Table 2:** Adiponectin-mediated outcomes and mechanisms in some preclinical cancer models.

Type of Cancer	Main Findings	Signaling Pathways	References
Breast	Adiponectin stimulates ERα+ MCF7 while inhibiting ERα− MDA-MB-231 cell proliferation	PI3K/AKT, MAPK	[76]
Lung	Adiponectin reduces viability and increases apoptosis in A549 cells	ERK1/2, AKT, CREB	[81,82]
	Adiponectin inhibits migration and invasion in NSCLC cells	Twist	[83]
Colorectal	Adiponectin represses colon cancer by inducing apoptosis	AMPK/mTOR, ERK1/2	[84,85,86]
	Adiponectin stimulates growth and inflammatory cytokine secretion in HT-29 cells	cAMP/PKA	[87]
	Adiponectin displays opposite effects in colorectal cancer depending on glucose availability	AMPK, PPARα, AKT	[88]
Thyroid	Adiponectin inhibits proliferation and migration of human papillary thyroid cancer cells	ULK/LC-3	[91]
Endometrial	Adiponectin impairs proliferation and induces apoptosis in human endometrial carcinoma cells	LKB1, AKT	[71,92]
	Adiponectin deficiency promotes endometrial carcinogenesis in PTEN-null mice	ERK1/2	[93]
Prostate	Adiponectin inhibits VEGF-mediated cancer neovascularization	AMPK/mTOR/VEGF	[95]

## 3. Adiponectin and Cell Metabolism

Adiponectin is a multifaceted protein that deeply regulates cell behaviors by affecting basal metabolic rate [96]. Through its endocrine and paracrine action, adiponectin improves insulin sensitivity and fatty acid oxidation [32]. Moreover, it also counteracts the detrimental effects of elevated intracellular ceramides [97]. With a special focus on glucose and lipid metabolism, the main alterations and the underlying mechanisms attributable to this adipose-derived hormone are discussed in this section.

### 3.1. Glucose Metabolism

As a stress-responsive kinase, AMPK plays an essential role in regulating glucose homeostasis [98]. Adiponectin, whose pleiotropic effects rely on AMPK at the cellular level, can directly perturb carbohydrate metabolism by acting on glucose trafficking, glycolysis, gluconeogenesis, and glycolytic disposal [99,100]. Unsurprisingly, adiponectin-induced AMPK phosphorylation has been reported in energy-intensive organs like the skeletal muscles, liver, and adipocytes [101,102,103].

The relevance of adiponectin to glucose homeostasis was elegantly demonstrated by knockout experiments in a mouse model for the first time [104]. Liu and collaborators highlighted that adiponectin deficiency causes the downregulation of essential glycolysis enzymes in hepatocytes, such as glucokinase, phosphofructokinase, and pyruvate dehydrogenase, as well as in tricarboxylic acid (TCA) enzymes. A slight increase was also observed for certain rate-limiting catalysts involved in gluconeogenesis. Concerning the glucose trafficking, globular adiponectin has been proven to increase its uptake in skeletal muscle cells via glucose transporter type 4 (GLUT4) translocation [105]. The newly internalized glucose was forced to pursue the anaerobic path, generating lactate as an end-product of glycolysis. Notably, glucose uptake and non-oxidative glycolysis are essential metabolic pathways for meeting the higher energy demands in skeletal muscle [106].

Related to glucose transportation and utilization, adiponectin displays a high affinity towards insulin-related signaling [107]. In skeletal muscle and adipocytes, glucose uptake is mediated by IRS 1/2 adaptor proteins after insulin stimulation [108]. Coincidentally, a significant association has been observed between IRS 1/2 levels and the development of insulin resistance [109]. Through its downstream PIK3/AKT and STAT3 pathways, adiponectin can upregulate IRS 1/2, sensitizing cells to insulin action [47,110]. The potential crosstalk between adiponectin and insulin was initially addressed in both obese and lipoatrophic mice [111]. In this study, Yamauchi and collaborators believe that decreased levels of adiponectin correlate with the onset of insulin resistance. Interestingly, they also observed that resistant patterns were reversed by the combination of physiological doses of both adiponectin and leptin but partially rescued by single adipokines. Subsequent reports further established the efficacy of adiponectin in sensitizing cells to insulin-mediated outcomes, affecting not only glucose uptake but also gluconeogenesis [32]. In this respect, whilst circulating adiponectin prevents de novo synthesis of glucose, the intracellular synthesized form would seem to exhibit the opposite effect. Onodera and colleagues have recently demonstrated that adiponectin overexpression increases gluconeogenesis-related genes in kidney tubular cells [112].

Conflicting results have also been reported in mammals relating to the effect of adiponectin on glycogen content. The accumulation of branched glucose polymer was thought to be unaffected by adiponectin in the past [113]. However, recent studies have found that adiponectin promotes the synthesis and accumulation of glycogen in hepatocytes, as well as in other kinds of cells [114].

### 3.2. Lipid Metabolism

Complete fatty acid oxidation into carbon dioxide involves multiple metabolic pathways, including β-oxidation, TCA, and oxidative phosphorylation [115]. Alterations occurring in any process of the oxidation chain results in specific pathological disorders [116].

However, fatty acid transport also plays a crucial task in lipid homeostasis. Trafficking of these molecules across the plasma membrane is made possible by either transporter proteins or scavenging receptors, such as cluster of differentiation 36 (CD36) and uncoupling protein 2 (UCP2) [117]. Interestingly, adiponectin typically improves lipid transport between two sides of a membrane, and this process has often been associated with fat burning in skeletal muscle [118]. However, in high-fat-diet (HFD)-fed mice, adiponectin supplementation also limited the intramuscular content of various free fatty acids, as well as several TCA intermediate metabolites [98]. Considering that β-oxidation is not efficiently coupled to the TCA cycle in an aberrant lipid state, the reduced accumulation of both acylcarnitine and acetyl-CoA in HFD-fed mice treated with adiponectin may indicate better coupling between these two subsequent metabolic pathways.

The β-oxidation is controlled by various genes whose expression is regulated by PPAR-α signaling [119]. Remarkably, adiponectin has been reported to increase the transcriptional activity of PPAR-α via AMPK and p38 MAPK activation [120]. The same insulin-sensitizing effects partially rely on the oxidation of fatty acids. Specifically, the activation of peroxisome proliferator-activated receptor gamma coactivator 1 alpha (PGC-1α) via Ca^2+^ and AMPK/SIRT1 enhances the expression of several molecules involved in fatty acid oxidation after adiponectin binding with AdipoR1 [121]. Always through AMPK stimulation, adiponectin causes the inactivation of acetyl-CoA carboxylases (ACCs), which, catalyzing the carboxylation of acetyl-CoA to malonyl-CoA, fosters oxidation while inhibiting the synthesis of new fatty acids [119,122].

Liver functions are predominantly accomplished by hepatocyte nuclear factors (HNFs). Among them, HNF4a is known to modulate the expression of sterol regulatory element-binding protein 1 (SREBP1) and carbohydrate-responsive element-binding protein 1 (ChREBP1), which are majorly involved in hepatic lipogenesis [123]. It has been observed that the expression of HNF4a is remarkably diminished in adiponectin knock-out mice [123].

Apart from fatty acids, adiponectin can control other classes of lipids, such as ceramide and cholesterol. Elevated intracellular ceramides are associated with reduced nutrient uptake, decreased insulin sensitivity, and increased apoptosis. Conversely, the ceramide component sphingosine exhibits opposite biological effects when phosphorylated into S1P. Adiponectin lowers ceramide content and drives the ceramide rheostat toward S1P-mediated outcomes [124]. ATP-binding cassette A1 (ABCA1) is fundamental for enabling cholesterol efflux towards high-density lipoprotein (HDL) [125]. Intriguingly, adiponectin induces the expression of Apo-A1 and ABCA1 and promotes the efflux from the plasma membrane to nascent HDL particles in hepatocytes [126,127]. Nonetheless, hypoadiponectinemia has been linked with lower size of low-density lipoprotein (LDL), decreased HDL and lipoprotein lipase (LPL) activity, and higher levels of triglycerides [128].

## 4. Adiponectin and Cancer Metabolism

Elucidation of the cancer-related hallmarks highlighted complex metabolic reprogramming exceeding the scope of enhanced glycolysis rate (Warburg effect). Pavlova and collaborators have recently defined a framework with six key metabolic features involving: (i) the aggressive acquisition and utilization of nutrients; (ii) the exploitation of alternative metabolic pathways when primary sources are limited; (iii) the utilization of acquired nutrients for energy production and macromolecule biosynthesis; (iv) elevated the nitrogen demand to support the synthesis of proteins and nucleic acids; (v) the dynamic regulation of gene expression to support metabolic shifts and fluctuations; and (vi) the intricate metabolic crosstalk with the tumor microenvironment, impacting both nutrient supply and waste removal [129]. This model portrays cancer cell metabolism as a highly adaptable and interconnected system, which substantially differs from the physiological one. Considering its dynamic role in modulating metabolism, the main alterations induced by adiponectin on cancer cell fate are discussed in this section.

### 4.1. Glucose Metabolism

The role of glycolysis in cancer development and treatment has received increasing attention over the years [130]. The most predominant metabolic feature of cancer cells is the reliance on glycolysis instead of mitochondrial oxidative phosphorylation, even in aerobic settings [131]. In contrast to the Warburg effect, some studies support the idea that adiponectin may enhance oxidative phosphorylation while suppressing glycolysis and lactate formation [132,133].

Lactate dehydrogenase A (LDHA) is a tumor-promoting enzyme that preferentially catalyzes the conversion of pyruvate to lactate [21,134]. Increased LDHA levels have been implicated in different cancer processes, such as the epithelial-to-mesenchymal transition and invasion/migration [135,136]. Conversely, LDHA impairment negatively affected tumor growth and metastasis formation [137]. A recent study disclosed that the secretome from murine colon carcinoma CT26 cells induces cachectic features in both murine and human adipocytes associated with metabolic alterations [138]. Herein, the upregulation of LDHA and higher lactate production were linked with a concomitant reduction in adiponectin expression mainly by STAT3 cascade.

Proteomic analysis of an xenograft tumor also demonstrated significant upregulation of the glycolytic pathway in adiponectin knockout mice [133]. Whilst lower levels of adiponectin accelerated tumor xenograft growth, incubation with the same adipose-derived hormone significantly reduced proliferation in LNCaP and PC-3 prostate cancer cells. Interestingly, the assessment of glycolytic genes, such as aldolase A (ALDOA), phosphoglycerate kinase 1 (PGK1), phosphoglycerate mutase 1 (PGAM1), and glucose transporter 1 (GLUT1), depicted the presence of a significant negative association between adiponectin and glycolysis in these cancerous cells.

Besides inhibiting both glycolysis and lactate production, adiponectin also reduces the expression of pyruvate dehydrogenase kinase 1 (PDK1) [21]. PDK1 physiologically inhibits the pyruvate dehydrogenase complex (PDC), which is responsible for converting pyruvate into acetyl-CoA [139]. Therefore, by decreasing PDK1 levels, adiponectin promotes the activity of PDC, facilitating the conversion of pyruvate into acetyl-CoA and enhancing oxidative phosphorylation [21,140].

The activation of AMPK is pivotal in mediating adiponectin-mediated outcomes on glucose homeostasis, even in cancer. In support of this claim, AMPK has been shown to negatively regulate the Warburg effect in lymphoma cells [141]. Besides accelerating MYC-induced lymphomagenesis, genetic ablation of the α1 catalytic subunit of AMPK enhanced glucose consumption and lactate production in this type of blood cancer. Remarkably, stabilization of the hypoxia-inducible factor-1α (HIF-1α) was required for AMPK-induced metabolic changes, since silencing of HIF-1α reversed the shift to anaerobic glycolysis, as well as biosynthetic and proliferative advantages conferred by AMPKα signaling.

### 4.2. Lipid Metabolism

By maintaining energy homeostasis and other cellular functions, altered lipid metabolism collectively aids cancer cells [142]. Adiponectin encompasses a wide range of activities impacting lipid metabolism at multiple levels [118].

As regards the neoplasms, adiponectin has been reported to decrease lipid uptake through suppressing CD36 and LDL receptor expression [143,144]. However, even though the tumor microenvironment is usually enriched in lipids, cancer cells exhibit an increased reliance on de novo lipogenesis, which confers long-term protection against free radicals and chemotherapeutics [142]. Consistent with this assumption, cancer cells often show upregulation of genes involved in fatty acid synthesis, such as SREBP-1, ACC1, fatty acid synthase (FASN), and ATP citrate lyase (ACLY) [145]. Remarkably, globular adiponectin significantly suppressed lipogenesis through the downregulation of SREBP-1- and FASN-related enzymes in breast cancer [105].

Previous findings also suggest a compensatory mechanism by which cancer cells could promote lipophagy in an attempt to restore adiponectin-induced ATP depletion. As a crucial process for intracellular lipid degradation, autophagy has also been linked to the breast cancer-suppressing effects of adiponectin [146]. Subsequent labeling with anti-LC3 antibody and Nile red confirmed the adiponectin-induced lipophagy in mammary tumors, which in turn triggers the release and oxidation of free fatty acids [147]. As a whole, this multi-level regulation results in net intracellular lipid losses, which may then affect membrane shape and lipid-based signaling networks [21].

Apart from serving as substrates for cellular fuel, indeed, fatty acids are the building blocks of essential cellular components and functions. In essence, fatty acid derivatives are required for post-transcriptional protein modifications, second messenger-mediated signal transmission, membrane structure, and fluidity maintenance [148]. Thus, it is not surprising that many cancer cells exhibit amplified dependence on fatty acid production and/or exogenous uptake. By degrading cell remnants, adiponectin triggers lipid raft disruption and signal transduction inhibition along the plasma membrane [147]. Notably, lipid rafts are largely influenced by both levels and composition of the cellular fatty acid [149]. Nevertheless, further studies are needed to fully understand the impact of adiponectin on non-metabolic lipids’ function.

As a leading regulator of adiponectin-mediated outcomes, AMPK activation exhibited significant inhibitory effects on gefitinib-resistant NSCLC cell lines along with perturbation in lipid metabolism [150]. Conversely, silencing of AMPK in both transformed and non-transformed cells resulted in a metabolic switch distinguished by an increased flux of glycolytic intermediates toward lipid biosynthesis [141]. The ability of adiponectin to regulate tumor growth is also aided by the impairment of the mTOR pathway, which is essential for maintaining lipid homeostasis [39,151]. By deactivating mammalian target of rapamycin complex 1 (mTORC1), for instance, adiponectin has been shown to inhibit SREBPs-dependent lipogenesis [21].

### 4.3. Metabolic Oxidation and Oxidative Stress

Even while cancer cells frequently exhibit a shift from oxidative phosphorylation to glycolysis, mitochondrial metabolism is gaining significance in terms of growth and survival [152]. Oxidative phosphorylation is essential for promoting the proliferation of malignant cells, even where glycolysis may supply enough ATP for cancer cell growth [153]. Analogously, the activation of oxidative phosphorylation and mitochondrial functions are required to develop both chemo and radio resistance [154].

Together with fatty acid oxidation, mitochondrial respiration represents the main intracellular source of free radicals [155]. Oxidative stress (OS) reflects an imbalance between the generation of ROS and antioxidant systems involving vital enzymes like superoxide dismutase (SOD), catalase, and manganese superoxide dismutase (MnSOD) [156]. Activating a variety of pro-carcinogenic processes, OS actively contributes to the onset, growth, and progression of neoplastic diseases [157].

Conflicting evidence exists regarding the influence of adiponectin on redox imbalance, causing either inhibitory or stimulatory effects in tumor cells. In endometrial cancer, for instance, adiponectin increased the cellular ROS levels, leading to apoptosis [92]. The inhibiting action of adiponectin on both the growth and migration of CaCo-2 and HCT116 colorectal cancer cells was largely counteracted by ROS-scavenging N-acetylcysteine [86].

The labeling of oxidative and nitrosative markers in tumor tissues revealed that exogenous adiponectin protects normal tissues from radiation-induced OS damage but has no effect on prostate cancer [158]. In accordance with these findings, several pieces of evidence emphasize the role of adiponectin in maintaining oxidative equilibrium in healthy cells [159,160,161]. The upregulation of nuclear factor erythroid 2-related factor 2 (NRF2), followed by a subsequent decrease in NADPH oxidase 2 (NOX2), defends normal compartments against OS when adiponectin signaling is enabled. Protection from ROS is not limited to healthy tissues, since comparable adiponectin-mediated features have been reported even in cancer models. Lu and coworkers demonstrated that adiponectin increases anti-oxidative defense while inhibiting OS in prostate cancer cells [162]. Specifically, adiponectin decreased the generation of superoxide anions, while increasing the transcript levels of both catalase and MnSOD in this hormone-dependent male cancer.

These findings pointed out the contribution of antioxidant mechanisms in determining redox status. The authors consciously corroborated adiponectin’s ability in stimulating ROS production. However, they proposed an experimental model in which adiponectin concurrently acts on the expression of antioxidant enzymes, thus suppressing OS levels in prostate cancer [162]. As widely known, cancer cells typically experience aberrant redox homeostasis, in contrast to normal ones. But while ROS are considered protumorigenic compounds, unsustainable ROS levels lead to cytotoxic effects even in tumor cells [163]. Therefore, a proper balance between ROS production and scavenging is also required to initiate and assist tumor progression. In conclusion, adiponectin can either promote or restrain OS depending on the redox state of each target cell.

## 5. Adiponectin Receptor Agonist AdipoRon in Cancer Metabolism

Identified through chemical library screening at the University of Tokyo in 2013, AdipoRon represents the first synthetic orally active adiponectin receptor agonist [164]. Since its discovery, AdipoRon has become attractive for its therapeutic potential, particularly in oncology. The first anticancer evidence was obtained in pancreatic ductal adenocarcinoma (PDAC), where AdipoRon demonstrated a strong inclination in inhibiting cancer cell proliferation [24,165].

Subsequently, the antineoplastic role of AdipoRon was also explored in other malignancies, such as NSCLC, osteosarcoma, prostate cancer, ovarian cancer, and cholangiocarcinoma [22,26,28,166,167]. The latest evidence also emphasizes the possibility of using AdipoRon as a potential partner in chemotherapy-based therapy, either to enhance therapeutic efficacy or overcome the occurrence of resistance [168,169,170].

As a key feature of the antineoplastic action, AdipoRon strongly perturbs cancer cell metabolism. The following subsections summarize the current understanding of AdipoRon-mediated effects on major metabolic pathways in tumor microenvironments.

### 5.1. Glucose Metabolism

**Figure 2:**
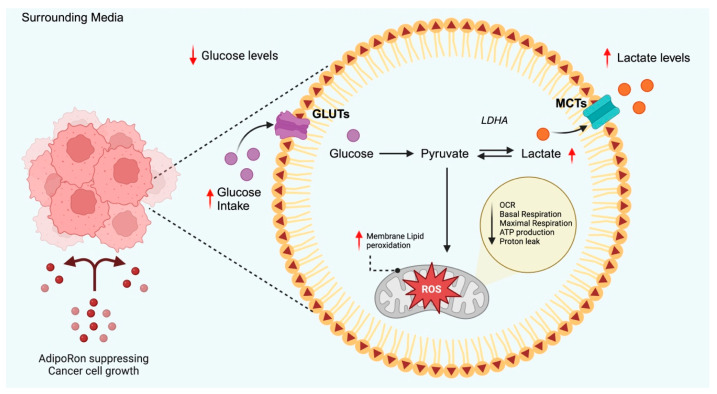
Schematic representation of AdipoRon-mediated effects on glucose metabolism proposed in PDAC. Current findings suggest that AdipoRon increases glucose uptake without any changes in GLUTs expression. This phenomenon involves an enhanced glycolytic rate that leads Acetyl-CoA to shift towards the anaerobic branch due to mitochondrial dysfunction. The red arrows represent the AdipoRon-mediated outcomes. GLUTs: glucose transporters, MCTs: monocarboxylate transporters, OCR: oxygen consumption rate, LDHA: lactate dehydrogenase A, ROS: reactive oxygen species. Created in BioRender (https://BioRender.com, accessed on 29 May 2025).

The hypothesis that AdipoRon could alter glucose metabolism arose from the acidification of culture media observed first in PDAC (Figure 2) and more recently in NSCLC [27,28]. Manley and coworkers revealed that AdipoRon significantly reduced the oxygen consumption rate (OCR) in PDAC cells [27]. Considering oxygen demand as an indicator of mitochondrial respiration, they proposed that potential mitochondrial impairment had occurred after AdipoRon treatment. To support this hypothesis, mitochondrial uncoupling and proton leak were subsequently assessed. Both metabolic mechanisms take part in the coupling of substrate oxygen to ADP phosphorylation/ATP generation. Hence, the increase in mitochondrial uncoupling and the concomitant decrease in proton leak were accepted as a further proof of the mitochondrial impairment in AdipoRon-treated PDAC cells.

As a compensatory mechanism for impaired oxidative phosphorylation, PDAC cells displayed an increased extracellular acidification rate (ECAR), which reflects enhanced glycolytic activity. Despite no changes being detected in GLUT1 and GLUT4 levels, AdipoRon treatment increased glucose utilization and lactate release into the conditioned media. Similar findings have recently been stated in NSCLC cells, where AdipoRon decreased glucose content and increased lactate accumulation in AdipoRon-exhausted cell culture media [28]. Supporting the hypothesis that glycolysis drives survival mechanisms to overcome mitochondrial dysfunction, in both studies, the exposure of AdipoRon-treated cells to glycolytic inhibitors produced a statistically significant decrease in cell growth [27,28].

At odds with these findings, Li and collaborators described that AdipoRon downregulates both RNA and protein levels of key glycolytic markers in thyroid cancer cells, such as GLUT1, pyruvate kinase M2 (PKM2), and LDHA [170]. Notably, AdipoRon demonstrated antiproliferative effects in this cancer type as well.

### 5.2. Lipid Metabolism

**Figure 3:**
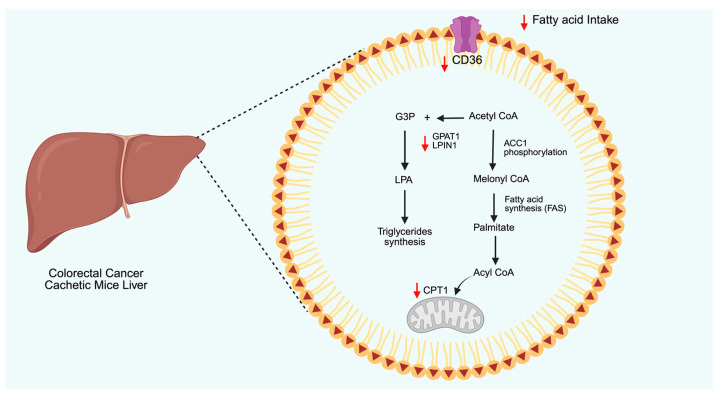
Schematic representation of AdipoRon-mediated effects on lipid metabolism reported in cachectic mice. AdipoRon reduces fatty acid intake by downregulating CD36. Simultaneously, AdipoRon affects fatty acid oxidation by inhibiting the entry of Acyl-CoA in mitochondria via carnitine palmitoyltransferase 1 (CPT1). AdipoRon also lowered the synthesis of triglycerides by downregulating the expression of glycerol-3-phosphate acyltransferase 1 (GPAT1) and lipin-1 (LPIN1) enzymes. The red arrows represent the AdipoRon-mediated outcomes. CD36: cluster of differentiation, LPA: lysophosphatidic acid, ACC1: acetyl-CoA carboxylase 1, GPAT1: glycerol-3-phosphate acyltransferase 1, G3P: glyceraldehyde 3-phosphate. Created in BioRender (https://BioRender.com, accessed on 29 May 2025).

The ability of AdipoRon to modulate lipid metabolism has lately been investigated in colorectal cancer, particularly in the context of cachexia (Figure 3) [171]. Specifically, AdipoRon decreased the transcript levels of the lipolysis enzymes in adipose tissue, while affecting triglyceride synthesis and fatty acid uptake in hepatocytes. As a result of combined whole-body lipid metabolism, AdipoRon significantly reduced hypertriglyceridemia, which in turn ameliorated the cachectic phenotype. Overall, attenuation of adipose triglyceride lipase (PNPLA2), as well as hepatic glycerol-3-phosphate acyltransferase 1 (GPAT1) and CD36, sustained the proposed experimental design in this study.

The activation of AMPK signaling leads to ACC1 phosphorylation, which in turn inhibits lipogenesis and promotes fatty acid oxidation. Despite AdipoRon stimulating both AMPK and ACC1, no significant enhancement in fatty acid oxidation was observed in PDAC cells due to the simultaneous appearance of mitochondrial dysfunction [27].

AdipoRon-mediated influences on cholesterol metabolism have also been reported in cancer models. In human colorectal adenocarcinoma, for instance, AdipoRon treatment decreased plasma membrane rigidity by reducing free cholesterol levels [172]. Since cholesterol is usually shuttled between different cellular organelles, the decrease in membrane content may reflect its redistribution to different compartments. Indeed, AdipoRon-treated cells showed the formation of free cholesterol-enriched lysosomes.

## 6. Other Adiponectin Receptor Agonists

Beyond AdipoRon, several other adiponectin receptor agonists have been identified and evaluated for their therapeutic potential.

ADP355 is a peptide-based adiponectin receptor agonist with very low nanomolar activity [77]. Initially studied for its antifibrotic and anti-inflammatory properties, ADP355 has also demonstrated antitumoral potential, particularly in prostate cancer cells [94]. Its antitumor features were correlated with altered cellular energetics, oxidative stress, and protein synthesis, as supported by functional gene set enrichment analysis. Fatty acid biosynthesis and mitochondrial dysfunction were the top-scoring metabolic pathways. As a master regulator of lipogenesis, SREBP1 protein levels trended lower in ADP355-treated tumors.

JT003 is a synthetic dual AdipoR1/R2 agonist, primarily evaluated in metabolic and fibrotic disease models. In non-alcoholic steatohepatitis (NASH), JT003 significantly reduced insulin resistance and inhibited hepatic stellate cell (HSC) activation in the liver [173].

GTDF, also known as GTDF-1, is a small-molecule flavonoid-based adiponectin receptor agonist, structurally related to quercetin. It has been shown to attenuate the diabetic phenotype and its complications in obese db/db mice through an AdipoR1-dependent mechanism [121].

Tiliroside, a plant-derived glycosidic flavonoid, also binds adiponectin receptors. Stimulation of fatty-acid oxidation has been reported in obese, diabetic mice treated with tiliroside [174].

## 7. Discussion

Starting from the assessment that adiponectin plays a crucial role in regulating cellular homeostasis, the existing evidence depicts an ambiguous framework with respect to either glucose or lipid metabolism.

Almost certainly, the most emblematic circumstance arises by comparing normal and cancer cells as regards glucose metabolism. Whilst adiponectin appears to promote glucose intake and utilization in healthy tissues, the same hormone-mediated stimulation counteracts the anaerobic production of ATP in cancer cells [104,110,133,138]. However, replacing adiponectin with its synthetic receptor agonists increases the cancer’s reliance on Walburg effects [27,28].

AdipoRon-related findings in particular could hint at a potential clarification of this metabolic discrepancy. AdipoRon has been proven to induce glycolytic dependence in both PDAC and NSCLC cells, by enhancing glucose consumption and lactate fermentation [27,28]. On the contrary, the mRNA levels of key genes involved in glucose homeostasis were downregulated in thyroid cancer cells treated with AdipoRon [167]. Considering that AdipoRon unveiled antineoplastic features in all the mentioned cancer models, its dissimilar metabolic effects could be due to the intrinsic aptitude of target cells for covering the energy demand from multiple sources. In this regard, the expression levels of adiponectin receptors could be critical in driving the metabolic balance towards glucose dependency, as well as increasing the abundance of certain adiponectin receptor subtypes relative to others.

The impairment of molecular effectors represents another substantial insight into elucidating the dual role of adiponectin regarding glucose metabolism. Required for adiponectin-mediated effects, LKB1 activates AMPK during energy stress to shift metabolic processes from anabolic to catabolic pathways [175]. LKB1 is spontaneously mutated in many cancers, including NSCLC, cervical, skin, and hepatocellular carcinoma [176]. Interestingly, different effects of adiponectin have been reported in cancer cells depending on LKB1 status. In breast cancer, for instance, treatment of stable LKB1-null MCF7 cells with adiponectin displayed no effects on both migration and invasion, while simulating LKB1-proficient MCF7 with the same adipose-derived hormone promoted their metastatic potential [177]. LKB1 depletion also prevented autophagy induction and growth inhibition in MDA-MB-231 breast cancer cells exposed to adiponectin treatments [146]. Although no cumulative findings support the influence of LKB1 on the intracellular fate of glucose, the inherited status of signaling pathways involved in adiponectin-mediated effects should be taken into consideration to deduce its metabolic outcome.

Certainly, cancer-related findings correlating glucose metabolism with adiponectin remains exceedingly limited, leaving space for misleading interpretations. The latest cancer evidence suggesting that adiponectin is an anti-glycolytic agent has been obtained from studies in knockout mice for the gene encoding this adipokine [133]. As recently stated, congenital deletion of adiponectin may induce potential adaptive responses that mask its biological phenotype [178]. Specifically, the onset of exhaustion could lead to hormonal imbalance, which in turn could affect glucose metabolism irrespective of adiponectin perturbation.

The emerging outcomes on lipid metabolism appear more consistent and reliable instead. The induction of β-oxidation and inhibition of lipogenesis are the key functions of adiponectin in either normal or cancerous tissue [121,122,146,147]. Comparable results have been reported regarding the reaction to the plant-derived agonist of adiponectin receptor tiliroside, although not in cancer [174].

But there is a peculiar facet of lipid metabolism in which healthy and cancer cells behave in ways opposite to adiponectin stimulation, namely, fatty acid intake. In contrast to normal tissues, both adiponectin and AdipoRon hinder the transport of fatty acids across the plasma membrane in the event of tumor growth [118,171,179]. Considering lipids as an essential constituent for tumor growth and dissemination, the lack of intake further supports the anticancer properties of adiponectin, as well as its synthetic receptor agonists. Another way to interpret this inconsistency lies in the function carried out by these membrane carriers. Besides modulating distribution and the passage of lipids across cell membranes, transport proteins also act as biological catalysts by accelerating specific chemical reactions [180]. Because many lipids function as second messengers, fatty acid transport proteins have also been implicated in cancer-associated signal transduction cascades. Notably, the effects of adiponectin on lipid intake are often assessed through the expression levels of transporter proteins rather than a proper evaluation of the fatty reserves in these studies. Thus, further efforts are required to clarify the involvement of transport proteins in adiponectin-mediated effects apart from the regulation of lipid trafficking.

## 8. Future Directions

Whilst a more comprehensive investigation is needed to recapitulate the involvement of adiponectin in cancer metabolic reprogramming, translating this knowledge from preclinical into human pathology will represent the real challenge in future.

If we want to use agonistic compounds as synthetic analogues of human adiponectin, pharmacokinetics and pharmacodynamics studies are required along with the assessment of their security profile. Currently, no clinical trials have been conducted to address either the safety or efficacy of adiponectin receptor agonists in oncology. The same AdipoRon, which has shown promising results in animal models, has only been used for research purposes to date [181,182]. However, within the same class of medications, ADP355 has been evaluated in a phase 1/2a trial involving subjects with xerophthalmia (NCT04201574). Besides revealing consistent improvements in symptoms, this adiponectin-based peptide was classified as a safe and well-tolerated medication [183]. Nonetheless, a subsequent phase 2b/3 trial is currently ongoing, involving almost 900 patients (NCT04899518). Adiponectin receptor agonists embrace a heterogeneous group of compounds, as supported by comparative structural analysis between ADP335 and AdipoRon (the most extensively tested molecules). Whilst ADP355 is an adiponectin-based short peptide distinguished by ten non-natural amino acids, AdipoRon exhibits three distinct functional groups arranged in the following order: a 1-benzyl 4-substituted 6-membered cyclic amine moiety, a carbonyl group, and a terminal aromatic ring [23,164]. Their different chemical nature implies conducting separate clinical trials, leading to an increase in costs and extended timelines for approval.

Despite the promising anticancer results achieved at the preclinical stage, metabolic drugs are often trapped in the early phases of clinical trials, experiencing either termination or suspension [184]. Originally proposed as a metabolic compound, metformin demonstrated anticancer effects in both in vitro and in vivo models. However, disappointing results have been attained in a number of large randomized clinical trials [185]. An increasing number of studies have also tested metformin in combination with conventional anticancer therapy, showing promising clinical benefits instead [186]. Besides sustaining cancer growth and progression, metabolic adaptations usually serve to overcome therapy-induced stress [184]. Therefore, targeting specific vulnerability could sensitize cancer cells towards standard-of-care therapies.

Synergistic potential has also been reported by combining AdipoRon with widely used chemotherapy agents [168,170]. Specifically, AdipoRon has been recognized as a potential candidate in gemcitabine-based therapy in PDAC cells [168]. Remarkably, integrating AdipoRon with gemcitabine was effective in constraining PDAC growth in resistant cells. Particularly, the AdipoRon-induced metabolic changes could support the ability to overcome resistance. Mitochondria have been described to sustain survival mechanisms in cancer cells treated with cytotoxic agents [187]. Since an impairment of oxidative phosphorylation has also been observed in response to AdipoRon administration, the damage occurring to this organelle could sensitize PDAC cells to gemcitabine [24,27]. More recently, AdipoRon has also been designated as effective support in combination with paclitaxel, expressly for NSCLC treatment [170]. However, metabolic engagement in AdipoRon-induced paclitaxel sensitization remains totally unexplored, representing an undoubtedly attractive field for future research studies.

Finally, we should take into account that prevention remains the first and most effective remedy against the onset of malignant disorders. Living a healthy lifestyle, which includes, among others, proper body weight control, is pivotal to preventing the occurrence of cancer. Incidentally, the negative effects of obesity on the prevalence of chronic diseases have been established over many years and recently confirmed by applying a semiparametric copula model [188]. Despite its multi-factorial and neuro-behavioral nature, balanced diet and regular exercise can restrain overweight and obesity [189]. In this intricate scenario, adiponectin plays a dynamic role as the most abundant adipose-derived hormone. Dietary factors can directly affect the serum concentrations of adiponectin other than calorie intake. Whilst monounsaturated and polyunsaturated fatty acids, fiber, polyphenols, alcohol, and milk lead to a marked increase in the blood levels of adiponectin, saturated fatty acids, saccharides, and red meat cause the opposite effects [190]. Additionally, a diet enriched in phytate, a natural compound that is predominantly abundant in cereals, legumes, and nuts, has demonstrated beneficial properties in patients with type 2 diabetes, either by reducing glycated hemoglobin or by increasing adiponectin levels [191]. Experimental and clinical data addressing the consequences of physical exercise on adiponectin levels are sometimes conflicting. Two extensive meta-analyses have revealed that exercise significantly increases the circulating levels of adiponectin [192,193]. In line with these findings, Mallardo and collaborators have recently confirmed improvements in adiponectin in relation to physical performance in young obese subjects [194]. However, there are also a number of studies that have failed to record adiponectin changes following physical activity [122]. Remarkably, the missing correlation appears to be pronounced in patients who have already experienced chronic diseases rather than in healthy subjects [195].

Could adiponectin be the magic bullet against cancer? Systematic reviews and meta-analyses might help in defining the pathogenic role of this adipose-derived hormone in neoplasms, as well as in other metabolic-related diseases.

## 9. Conclusions

In this review, we assessed the impact of adiponectin on cancer metabolic reprogramming, focusing mainly on the intracellular fate of both glucose and lipids. Conflicting and contradictory reports are currently available with respect to adiponectin-mediated outcomes between cancer and normal cells. Certainly, the regulation of glucose and lipid homeostasis contributes to the antitumor effects induced by this adipokine, even though adiponectin-based therapy is considered unsuitable due to intrinsic limitations.

The discovery of small and synthetic adiponectin receptor agonists has provided new therapeutic insights in cancer treatment. Among them, AdipoRon has emerged as a potent anticancer agent affecting cancer metabolic reprogramming. Regulatory features have been assigned to AdipoRon concerning glycolysis, fatty acid oxidation, and de novo synthesis. Nevertheless, further insights into the role of both adiponectin and adiponectin receptor agonists are needed to develop tailored therapies for targeting metabolic pathways in cancer management.

## Figures and Tables

**Table 1:** Circulating adiponectin and cancer: supporting and conflicting evidence among various types of malignancies.

Type of Cancer	Supporting Data	Conflicting Data
Breast	Lower adiponectin is associated with a higher risk of breast cancer in postmenopausal women [60,61]	Circulating adiponectin is not related to breast cancer risk in premenopausal women [61]
Lung	Adiponectin levels are reduced in lung adenocarcinoma, as well as in advanced patients [62,63]	Bloodstream adiponectin in lung cancer does not differ from that in healthy subjects [62,64]
Colorectal	Colorectal cancer cases exhibit diminished adiponectin than those without malignancy [65,66]	Serum adiponectin is not associated with the risk of colorectal cancer [67]
Thyroid	Adiponectin is inversely correlated with the risk of thyroid cancer, expressly in women [68]	Thyroid carcinomas and controls display comparable adiponectin levels in blood [69]
Endometrial	Lower adiponectin levels increase the risk of endometrial cancer and correlate with its grade [70,71]	Plasma concentration of adiponectin is not predictive of endometrial cancer risk [72]
Prostate	Reduced adiponectin expression is linked with a higher degree and stage of prostate cancer [73]	Adiponectin unveils a weak correlation with respect to prostate cancer aggressiveness [74]

## Data Availability

Not applicable.

## Abbreviations

The following abbreviations are used in this manuscript:

AMPK	AMP-activated protein kinase
AICAR	5-aminoimidazole-4-carboxamide-1-β-d-ribofuranoside
BMI	Body mass index
LMW	Low molecular weight
MMW	Medium molecular weight
HMW	High molecular weight
AdipoR1	Adiponectin receptor 1
AdipoR2	Adiponectin receptor 2
ECs	Extracellular domains
PPAR-α	Peroxisome proliferator-activated receptor alpha
APPL1/2	Phosphotyrosine interacting with PH domain and leucine zipper 1/2
IRS 1/2	Insulin receptor substrate proteins 1/2
PP2A	Protein phosphatase 2A
LKB1	Liver kinase B1
PKCζ	Protein kinase Cζ
CaMKK	Calmodulin-dependent protein kinase
PLC	Phospholipase C
IP3	Inositol triphosphate
ER	Endoplasmic reticulum
SIRT1	Sirtuin 1
mTOR	Mammalian target of rapamycin
MAPK	Mitogen-activated protein kinase
PI3K	Phosphatidylinositol 3-kinase
AKT	Protein kinase B
NF-κB	Nuclear factor kappa B
JAK	Janus kinase
STAT	Signal transducer and activator of transcription
IκBα	Inhibitor of nuclear factor kappa B
PKA	Protein kinase A
NO	Nitric oxide
ROS	Reactive oxygen species
S1P	Sphingosine 1-phosphate
ERα	Estrogen receptor alpha
NSCLC	Non-small-cell lung cancer
CREB	Cyclic AMP-responsive element-binding protein
ERK 1/2	Extracellular signal-regulated kinase 1/2
RB	Retinoblastoma-associated protein
USP2	Ubiquitin carboxyl-terminal hydrolase 2
BCL-2	B-cell leukemia/lymphoma 2
PTEN	Phosphatase and tensin homolog
VEGF	Vascular endothelial growth factor
TCA	Tricarboxylic acid cycle
GLUT4	Glucose transporter type 4
CD36	Cluster of differentiation 36
UCP2	Uncoupling protein 2
HFD	High-fat diet
PGC-1α	Peroxisome proliferator-activated receptor gamma coactivator 1 alpha
ACC	Acetyl-CoA carboxylase
HNF	Hepatocyte nuclear factor
SREBP1	Sterol regulatory element-binding protein 1
ChREBP1	Carbohydrate-responsive element-binding protein 1
ABCA1	ATP-binding cassette A1
HDL	High-density lipoprotein
LDL	Low-density lipoprotein
LPL	Lipoprotein lipase
LDHA	Lactate dehydrogenase A
ALDOA	Aldolase A
PGK1	Phosphoglycerate kinase 1
PGAM1	Phosphoglycerate mutase 1
GLUT1	Glucose transporter 1
PDK1	Pyruvate dehydrogenase kinase 1
PDK	Pyruvate dehydrogenase complex
HIF-1α	Hypoxia-inducible factor 1α
FASN	Fatty acid synthase
ACLY	ATP citrate lyase
mTORC1	Mammalian target of rapamycin complex 1
OS	Oxidative stress
SOD	Superoxide dismutase
MnSOD	Manganese superoxide dismutase
NRF2	Nuclear factor erythroid 2-related factor 2
NOX2	NADPH oxidase 2
PDAC	Pancreatic ductal adenocarcinoma
OCR	Oxygen consumption rate
ECAR	Extracellular acidification rate
PKM2	Pyruvate kinase M2
MCT	Monocarboxylate transporter
PNPLA2	Adipose triglyceride lipase
GPAT1	Glycerol-3-phosphate acyltransferase 1
CPT1	Carnitine palmitoyltransferase 1
LPIN1	Lipin-1
LPA	Lysophosphatidic acid
G3P	Glyceraldehyde 3-phosphate
NASH	Non-alcoholic steatohepatitis
HSC	Hepatic stellate cell
